# Liquid–liquid phase separation in hepatocellular carcinoma

**DOI:** 10.3389/fcell.2024.1454587

**Published:** 2024-12-24

**Authors:** Jianguo Xu, Wangwang Liu, Yihan Yao, Tuomas P. J. Knowles, Zhi-Gang Zhang, Yan-Li Zhang

**Affiliations:** ^1^ State Key Laboratory of Systems Medicine for Cancer, Shanghai Cancer Institute, Ren Ji Hospital, School of Medicine, Shanghai Jiao Tong University, Shanghai, China; ^2^ Yusuf Hamied Department of Chemistry, University of Cambridge, Cambridge, United Kingdom

**Keywords:** liquid-liquid phase separation, hepatocellular carcinoma, cytoplasmic condensates, nuclear condensates, droplets

## Abstract

Liquid-liquid phase separation (LLPS) drives the formation of membraneless intracellular compartments within both cytoplasm and nucleus. These compartments can form distinct physicochemical environments, and in particular display different concentrations of proteins, RNA, and macromolecules compared to the surrounding cytosol. Recent studies have highlighted the significant role of aberrant LLPS in cancer development and progression, impacting many core processes such as oncogenic signalling pathways, transcriptional dysregulation, and genome instability. In hepatocellular carcinoma (HCC), aberrant formation of biomolecular condensates has been observed in a number of preclinical models, highlighting their significance as an emerging factor in understanding cancer biology and its molecular underpinnings. In this review, we summarize emerging evidence and recent advances in understanding the role of LLPS in HCC, with a particular focus on the regulation and dysregulation of cytoplasmic and nuclear condensates in cancer cells. We finally discuss how an emerging understanding of phase separation processes in HCC opens up new potential treatment avenues.

## Introduction

LLPS is a common form of density transition that many macromolecules can undergo, where a homogeneous solution spontaneously decomposes into a solute depleted phase and a solute rich phase. The importance of LLPS for functional biology was initially introduced by Hyman and Brangwynne in 2009. This pivotal observation demonstrated that P granules, responsible for RNA and protein’s regulation, display liquid-like properties, thus highlighting the importance of dense liquid phases in functional biology. Different techniques, including live-cell imaging, genetic manipulation, and fluorescence recovery after photobleaching (FRAP) revealed a range of prototypical liquid-like behaviours such as fusion, dripping, and wetting (Brangwynne et al., 2009; Shin et al., 2017). It has become apparent that LLPS of this type is in fact encountered commonly in biology and serves as a mechanism for achieving spatiotemporal control, allowing for the compartmentalisation of biological reactions within distinct subcellular locations (Zhang et al., 2015). These findings indicate that the control of spatial localisation of biological molecules within a cell is not exclusively achieved by conventional membrane structures, but that the intrinsic phase behaviour of the component molecules is a crucial factor. Indeed, such membraneless processes exist to exert spatiotemporal control at subcellular and suborganelle levels, thus broadening our understanding of cellular regulation (Sabari et al., 2020). Furthermore, studies have revealed that dysregulation of LLPS plays a significant role in human health and diseases including Alzheimer’s disease (Boyko and Surewicz, 2022; Fu et al., 2024), Amyotrophic lateral sclerosis (Conicella et al., 2016), and notably cancer (Boija et al., 2021; Tong et al., 2022; Xie et al., 2023). Biomolecular condensates, formed through LLPS, are of considerable importance in directly regulating essential cellular processes associated with cancer cell pathology. For instance, mutations that affect the assembly behaviour of oncoproteins or tumour suppressors can lead to the formation of aberrant condensates that promote cancer progression. LLPS, driven by multivalent weak interactions among macromolecules, is fundamental to the formation of biomolecular condensates. Various combinations of these multivalent interactions can facilitate liquid-liquid phase transitions (LLPT) (Boeynaems et al., 2018; Taniue and Akimitsu, 2022). LLPT represents a cooperative process arising from the collective behaviour of interacting modules within multivalent proteins. Dysregulation of LLPS and LLPT leads to aberrant condensate and amyloid formation, which causes many human diseases such as neurodegeneration and cancer (Boeynaems et al., 2018; de Oliveira et al., 2019). Especially, the aberrant LLPS is increasingly recognized as a hidden driver of oncogenic activity (Jiang et al., 2020; Cai et al., 2021).

Recent studies have underscored the pivotal role of aberrant LLPS in tumour biology. Tumour cells frequently adapt their intracellular milieu to foster growth and dissemination. The dynamic modulation of LLPS can profoundly impact tumour cell behaviours by influencing gene expression patterns, protein functionality, and intracellular signalling pathways (Wang et al., 2021; Tong et al., 2022). Disruptions in normal LLPS processes, stemming from genetic or epigenetic mutations, can induce aberrant biomolecular condensates. These condensates contribute to tumourigenesis by affecting chromosome organization (Gibson et al., 2019), signal transduction (Zhang J. Z. et al., 2020), and transcriptional regulation (Han et al., 2020), thereby facilitating cancer development. Ming et al. demonstrated that cancer cells could be particularly sensitive to disruptions in LLPS, given the numerous abnormal biological processes inherent to cancer cells (Ming et al., 2019). The importance of LLPS is also being more widely recognized in physiology, especially in gastrointestinal cancers. For example, in oesophageal cancer, research has revealed that the MALR binds to the dsRBD1 domain of interleukin enhancer-binding factor 3 (ILF3), thereby enhancing the stability of ILF3 protein. This interaction promotes ILF3-mediated LLPS, which, in turn, increases HIF1α mRNA stability by preventing PARN-mediated degradation (Liu J. et al., 2023). In pancreatic ductal adenocarcinoma (PDAC), the KMT2D protein, with two low-complexity domain (LCD) structures, undergoes LLPS to regulate histone monomethylation transcription. This process is crucial for tumour cell proliferation and migration, facilitating PDAC progression (Li et al., 2021). Notably, stress granules (SGs) inhibit apoptosis and enhance PDAC viability. KRAS-mutated PDAC upregulates SGs levels, which is advantageous for PDAC development. Reagents that target SGs by blocking SG assembly pathways or promoting SG clearance have shown anti-tumour effects (Xing et al., 2023). More specifically, in colorectal cancer, treating colorectal cancer cells with MS-444, a small molecule targeting HuR, inhibits proliferation (Blanco et al., 2016). The phase separation ability of Axin facilitates the nuclear translocation of β-catenin and its incorporation into transcriptional condensates, impacting the transcriptional activity of the Wnt signalling pathway (Zhang et al., 2024). MiR-490-3p targets CDK1 through an LLPS-dependent miRISC system, inhibiting colon cancer cell proliferation. Furthermore, studies have revealed that SENP1 reduces RNF168 phase separation, promoting DNA damage repair and drug resistance in colon cancer (Da et al., 2019; Qin et al., 2021). These cases collectively highlight the significant role of LLPS in regulating the tumour procession within the digestive system.

Hepatocellular carcinoma (HCC), the predominant type of primary liver cancer, presents a significant global health challenge with a rising prevalence worldwide (Villanueva, 2019; Llovet et al., 2021). The World Health Organization predicts over 1 million deaths from liver cancer by 2030 (Organization, 2019). Globally, liver cancer ranks 6th in incidence (4.3%) and 3rd in mortality (7.8%). Primary liver cancer includes HCC (comprising 75%–85% of cases) and intrahepatic cholangiocarcinoma (10%–15%) and other rare types (Bray et al., 2024). HCC is a highly heterogeneous disease and poses significant challenges in prevention, diagnosis, and treatment (Llovet et al., 2021). Most HCC cases occur in individuals with underlying liver disease, often resulting from hepatitis B or C virus (HBV or HCV) infection or alcohol abuse (Villanueva, 2019). HCC poses a particularly challenging treatment landscape, as evidenced by its current heterogeneity and the limited efficacy of therapeutic responses (Yang et al., 2023). Hence, a comprehensive understanding of the mechanisms underlying HCC is essential for developing effective management strategies to address this escalating health concern. Several studies have highlighted the key role of LLPS in shaping the tumour microenvironment and influencing various aspects of HCC, including its initiation, development, progression, invasion and metastasis (Liu et al., 2021; Liu B. et al., 2023; Meng et al., 2023; Xiao et al., 2023). In this review, we classify HCC-related condensates into cytoplasmic, nuclear, and combined categories, based on the biophysical characteristics of cells, given that the presence of condensates on the nuclear or cell membrane remains unexplored. In Table 1, we summarize molecules related to LLPS in HCC. We aim to provide a comprehensive summary of the latest research on LLPS and its association with the initiation, progression, and metastasis of HCC, with the ultimate goal of shedding light on potential therapeutic strategies against HCC.

**TABLE 1:** LLPS-associated molecules in HCC.

Molecules	Condensates	Localisations	Functions related HCC	Refs
TAK1	TAK1-TAB3 bodies	Cytoplasm	1. Fetal TAK1 is constitutively active and forms liquid-like condensates with TAB3 in HCC cells	Yan et al. (2023)
YTHDF2	P granules	Cytoplasm	1. m6A RNA might promote YTHDF2-mediated phase separation 2. YTHDF2 promotes the CSC liver phenotype and cancer metastasis by modulating the m6A methylation of OCT4 mRNA.	Wang et al. (2020), Zhang et al. (2020a), Wang and Lu (2021)
Ferritin	URB1-AS1-ferritin complex	Cytoplasm	1. The URB1-AS1−Ferritin interaction promotes ferritin phase separation and inhibits NCOA4-mediated ferritin degradation	Gao et al. (2023)
p62	p62 bodies	Cytoplasm	1. p62-positive Mallory-Denk bodies are a prominent feature of HCC, and their biogenesis depends on the phase separation of p62 2. MOAP-1 interferes with the liquid–liquid phase separation of p62	Tan et al. (2021), Kurusu et al. (2023)
PKA	PKA RIɑ bodies	Cytoplasm	1. A PKA fusion oncoprotein associated with an atypical liver cancer potently blocks RIα LLPS and induces aberrant cAMP signalling	Zhang et al. (2020b)
CAPRIN1	Stress granule (SG)	Cytoplasm	1. Downregulate the protein level of Myc proto-oncogene protein by inhibiting c-Myc translation in hepatocellular carcinoma	Chen et al. (2022)
Glycogen	Glycogen-Laforin-Mst1/2 complex	Cytoplasm	1. Accumulated glycogen undergoes phase separation to suppress Hippo signalling and drive liver tumour initiation	Liu et al. (2021)
YAP/TAZ	YAP-TEAD complex	Nucleus	1. Facilitated transcription of oncogenic genes via LLPS to recruit the crucial transcription factor TEAD4 in hepatoblastoma	Mao et al. (2023)
SFPQ/Smad4	paraspeckle	Nucleus	1. SFPQ undergoes LLPS through its prion-like domain in HCC. 2. SFPQ suppresses TGF-b-induced growth-inhibitory and transcriptional responses	Xiao et al. (2023)
BRD4	Nuclear body	Nucleus	1. BRD4S forms discrete condensates in chromatin in liver cancer cells while the specific mechanism is unclear	Han et al. (2020)
INCENP, Borealin, Survivin, Aurora kinase B	Chromosome passenger complex	Nucleus	1. MLL1 methylates Borealin K143 and regulates LLPS of the CPC in mitotic cells, and CPC-high HCC shows elevated dependency on MLL1	Sha et al. (2023)
Pinin	Nuclear body	Nucleus	1. Pinin induces EMT by regulating m6A modification in HCC.	Qiao et al. (2021)
YBX1	Nuclear protein granule	Nucleus	1. CircASH2 facilitates the LLPS of nuclear YBX1 and targets TPM4 transcripts by assembling a complex with hnRNPs in HCC.	Liu et al. (2023a)
SURF6	NPM1-SURF6 complex	Nucleus	1. SURF6interacts with NPM1 playing a role in dynamic switching during phase separation	Qiu et al. (2015), Ferrolino et al. (2018)
Twist1,YY1	Twist1-YY1-p300 complex	Nucleus	1. Twist1 and YY1 can be affected by co-activator P300 2. Twist1/YY1/p300 phase separation complex promotes EMT in HCC by directly regulating the expression of miR-9	Ghanawi et al. (2021)
MAZ,CCND1,G4	MAZ-CCND1-G4 condensates	Nucleus	1. G4s recruit MAZ to the CCND1 promoter and facilitate the motility in MAZ condensates that compartmentalize coactivators to activate CCND1 expression and subsequently exacerbate hepatocarcinogenesis	Wang et al. (2024)
STAT3	STAT3 bodies	Nucleus and Cytoplasm	1. IL-6-activated STAT3 transcription factors form phase-separated biomolecular condensates in the cytoplasm and the nucleus	Sehgal (2019)

## Emerging evidence of LLPS in HCC cell cytoplasm

**FIGURE 1:**
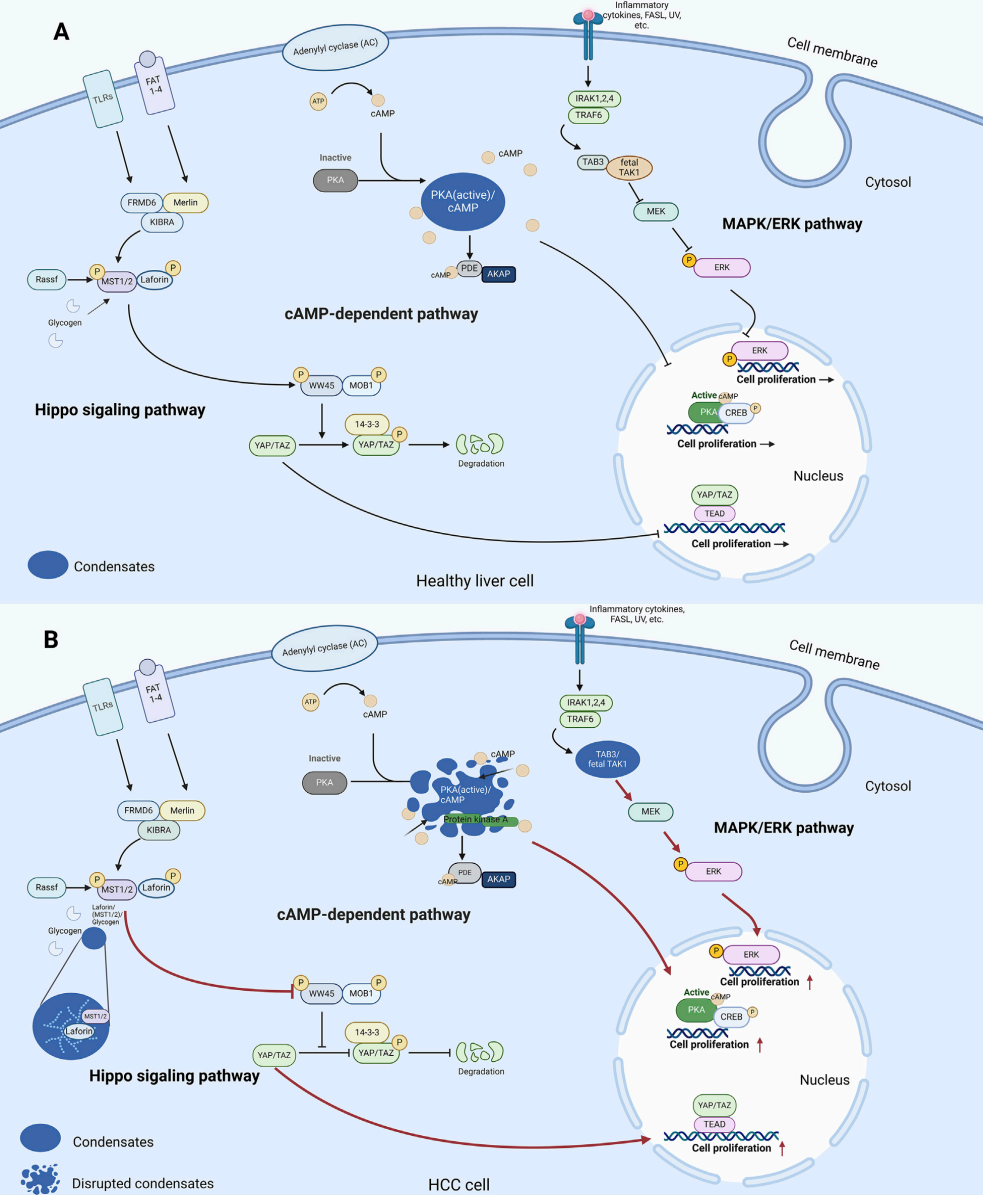
LLPS and signalling pathways in the cytoplasm of healthy liver cells and HCC cells. **(A)** In healthy liver cells, LLPS is involved in regulation of the cAMP-dependent pathway. In Hippo signalling pathway, the core kinases MST1/2 bind to Laforin, which then phosphorylate the transcription co-activators YAP and TAZ. Phosphorylated YAP/TAZ are sequestered in the cytoplasm and degraded, reducing their activity in promoting cell proliferation and growth. In the cAMP-dependent pathway, elevation of cAMP levels activates PKA. PKA then phosphorylates transcription factors CREB, ultimately regulating gene expression and cellular proliferation. In the MAPK/ERK pathway, TAB3 acts as an adaptor protein that recruits TAK1, facilitating its activation. Activated TAK1 then phosphorylates and activates downstream MAPK kinases, leading to the activation of ERK. **(B)** In HCC cells, LLPS regulates the transmission of three signalling pathways to regulate cell proliferation as follows. In the MAPK/ERK pathway, TAK1 forms liquid-like condensates with TAB3 to promote downstream signalling. In the cAMP-dependent pathway, the disputation of PKA/cAMP condensates disrupts cAMP compartmentation and leads to increased cell proliferation. In the Hippo signalling pathway, glycogen accumulates to form droplets, which promotes complex formation between Laforin and MST1/2, leading to tumourigenesis in a Yap-dependent manner (Red arrows in Figure 1B indicate changes compared to Figure 1A). Abbreviations: TLR5: Toll-Like Receptor 5, ATP: Adenosine Triphosphate, AMP: Cyclic Adenosine Monophosphate, PKA: Protein Kinase A, PIP2: Phosphatidylinositol 4,5-bisphosphate, IRAK1,2,4: Interleukin-1 Receptor-Associated Kinase 1, 2, 4, CREB: cAMP response element-binding protein, TRAF6: TNF Receptor-Associated Factor 6, TAK1: Transforming growth factor beta-Activated Kinase 1, MEK: Mitogen-activated protein kinase kinase, ERK: Extracellular signal-Regulated Kinases, MKK3: Mitogen-activated protein Kinase Kinase 3, MST1/2: Mammalian STE20-like protein kinase 1/2, LATS1/2: Large Tumor Suppressor kinase 1/2, YAP/TAZ: Yes-Associated Protein/Transcriptional coActivator with PDZ-binding motif, TEAD: TEA Domain transcription factor.

To explore how LLPS contributes to HCC tumourigenesis and unravel the intricate workings of intracellular signalling in the cytoplasm, we delve into key pathways including the MAPK, cAMP-dependent, and Hippo signalling pathways, which are key pathways that control cell proliferation, differentiation, and apoptosis in normal and tumour cells (Figure 1A). Moreover, we shed light on the importance of LLPS in regulating these pathways and its profound impact on cancer progression. Additionally, our analysis extends to cellular quality control mechanisms such as ferroptosis and autophagy, meticulously examining how LLPS regulates these processes and their implications in tumourigenesis. Furthermore, we investigate the dysregulation of epigenetic factors and the role of RNA in LLPS and HCC, providing a comprehensive understanding of the complex molecular landscape of HCC related to LLPS.

### LLPS and intracellular signalling pathways

LLPS and dysregulated intracellular signalling are closely related. One such pathway is the Mitogen-Activated Protein Kinase (MAPK) pathway, which plays a key role in transmitting and coordinating signals from different stimuli to elicit specific physiological responses in mammalian cells. These responses encompass cellular proliferation, differentiation, development, inflammatory reactions, and apoptosis in the healthy liver (Zhang and Liu, 2002) (Figure 1A). The MAPK pathway consists of four distinct signalling families that undergo a series of regulatory events within the cytoplasm (Burotto et al., 2014). These events exhibit precise spatial and temporal dynamics to effectively modulate the output of the MAPK/ERK pathway. Recent studies have shed light on the impact of LLPS on MAPK activation (Figure 1B). For instance, oncogenic mutations in SHP-2 have been found to facilitate LLPS, resulting in localized dephosphorylation events and subsequent MAPK signalling activation (Zhu et al., 2020 ). Moreover, SHP-2 operates as a critical mediator of hepatocyte-driven tumorigenic signalling downstream of receptor tyrosine kinases (RTKs) and its deficiency induces a tumour promoting hepatic microenvironment (Han et al., 2015; Liu et al., 2018). In HCC, fetal TGF-β Activated Kinase 1 (TAK1) is constitutively active and forms liquid-like condensates with TAK1-binding protein 3 (TAB3) within the cytoplasm of HCC cells. This interaction and subsequent condensate formation are hypothesized to sustain the activation of downstream signalling pathways in HCC (Yan et al., 2023).

Emerging evidence suggests that LLPS contributes to liver tumour development by influencing the Cyclic Adenosine Monophosphate (cAMP) pathway. In healthy liver cells, cAMP, a modulator of critical physiological processes such as metabolism, gene expression, and apoptotic pathways, is synthesized and broken down by Phosphodiesterases (PDEs) (Conti, 2000; Beavo and Brunton, 2002). Its association with Protein Kinase A (PKA), a tetrameric holoenzyme consisting of a regulatory subunit dimer bound to a pair of catalytic subunits through its cooperation with and the protein cAMP response element-binding protein (CREB) activates the kinase, enabling it to exert its effects on different cellular components and functions (Lonze and Ginty, 2002) (Figure 1A). The coordinated actions of cAMP and PKA play a critical role in regulating important processes such as cell growth and survival. Recent findings indicate that LLPS is involved in liver tumour development through the modulation of this pathway in response to dynamic cAMP signalling, the regulatory subunit Iα (RIα) of PKA undergoes LLPS at endogenous levels (Zhang J. Z. et al., 2020). This leads to the formation of specialized compartments called RIα bodies, which contain high levels of cAMP and PKA activity. This dynamic sequestration of cAMP is essential for spatially constraining its distribution within untransformed cells. RIα phase separation is critical for effective cAMP compartmentations which prevent tumourigenesis (Figure 1B). Fibrolamellar carcinoma (FLC) is a rare variant of HCC, comprising 1%–9% of all HCCs. In one of atypical liver cancers FLC, aberrations in the cAMP-dependent pathway occur through the disruption of LLPS (Honeyman et al., 2014). A PKAcat fusion oncoprotein can associate with PKA and disrupts RIα LLPS, leading to abnormal cAMP signalling. This loss of RIα LLPS in untransformed cells is associated with increased cell proliferation and cellular transformation. Therefore, spatially dysregulated cAMP/PKA pathway and disrupted RIα punctum leads to defective cAMP compartmentation, and the aberrant cAMP/PKA signalling caused by altered cAMP compartmentation is linked to this liver tumour (Zhang J. Z. et al., 2020).

Hippo pathway serves as an important tumour suppressor mechanism, with extensive research supporting its role in inhibiting tumour growth (Pan, 2010; Harvey et al., 2013). The Hippo pathway is a critical regulator of cell proliferation and apoptosis. Glycogenolysis enzymes, deficiency results in glycogen storage diseases (GSDs) associated with Yes-associated protein (YAP) activation, phenocopying Hippo signalling deficiency within the healthy liver (Figure 1A) (Wilson et al., 2019). In the context of cancer, glycogen dysregulation leads to the malignant transformation. For instance, the loss of STK3/STK4 which activates a kinase cascade that inhibits Yes-associated protein/Transcriptional coactivator with PDZ-binding motif (YAP/TAZ) effector proteins, controlling transcription related to cell growth in mouse liver models leads to uncontrolled cellular proliferation and differentiation, while overexpression of YAP/TAZ results in tissue hyperplasia and tumour formation (Camargo et al., 2007; Zhou et al., 2009). Interestingly, recent discoveries have uncovered a link between the formation of glycogen LLPS and liver tumourigenesis. In the context of HCC, glycogen accumulates to form droplets at high concentration, leading to the formation of complexes between Laforin, the Hippo kinases and mammalian STE20-like protein kinase 1/2 (MST1/2) that were encapsulated by the droplets. This process sequesters Mst1/2 and alleviates their inhibitory effect on YAP. Inactivation of the oncoprotein YAP by the Hippo/Mst1/2 pathway emerges as a critical tumour suppression mechanism (Figure 1B). Furthermore, deficiencies in G6PC or the glycogenolysis enzyme liver glycogen phosphorylase (PYGL) in both humans and mice result in glycogen storage disease. Under conditions of glycogen accumulation in live cells are undergoing LLPS which is associated with liver enlargement and tumourigenesis in a YAP-dependent manner. Consistently, reducing glycogen accumulation has been observed to inhibit liver growth and cancer development, while increased glycogen storage is linked to accelerated tumour formation. In view of the existing studies of LLPS in the pathogenesis of HCC, LLPS exacerbates pre-existing oncogenic effects. Whether it is the formation or destruction of condensates, LLPS plays a key role in the abnormal conditions of cell proliferation (Liu et al., 2021).

### LLPS and cellular quality control

Ferroptosis, a form of programmed cell death distinct from apoptosis, necrosis, and autophagy, has emerged as an important tumour suppression mechanism and is characterized by the iron-dependent accumulation of reactive oxygen species (ROS) to lethal levels (Chen et al., 2021). Most recently, LLPS has been implicated in the clearance of cancer cells through its dysregulation of ferroptosis. For example, LLPS promotes the binding of FASA, a long non-coding RNA (LncRNA), to the Ahpc-TSA domain of Peroxiredoxin 1 (PRDX1) and inhibits its peroxidase activity, thus disrupting intracellular ROS balance. In addition, LLPS facilitates the degradation of Ferritin, an important protein involved in the storage of iron, which prevents ferroptosis. Thus, in regulating the ferroptosis pathway, LLPS is involved in activation-related activities of cancer (Chen et al., 2021).

Specifically in HCC, it has been demonstrated that the induction of phase separation of Ferritin by the direct interaction with lncRNA URB1-AS1, can reduce intracellular free iron levels and inhibit ferritinophagy triggered by the chemotherapy drug sorafenib. Since Ferritin degradation is normally initiated by binding with the Nuclear Receptor Coactivator 4 (NCOA4) complex, the formation of LLPS between URB1-AS1 and Ferritin inhibits its degradation. This mechanism is achieved, in part, by the formation of a cluster formed from the encapsulated lncRNAs, and various recruited RNA and proteins together with URB1-AS1 and ferritin. These clusters block direct interaction between the NCOA4 and the ferritin by steric hindrance and prevent the ferritin clusters from being transported to lysosomes for their degradation. As ferritin accumulates (ferritinophagy), iron becomes trapped, causing functional iron deficiency. Excessive levels of iron inside cells are harmful, which can trigger a chain reaction that damages cell membranes leading to cell death by ferroptosis (Gao et al., 2023).

Beyond ferroptosis, LLPS as also has an established role as positive regulator of autophagic activity through diverse mechanisms. Notably, phase separation plays a crucial role in modulating the activity of the target of Rapamycin Complex 1 (TORC1): the suppression of TORC1 signalling triggers the initiation of autophagy, and dysregulation of both TORC1 and autophagy has been implicated in the development of tumours (Noda et al., 2020).

Recent research has highlighted the significance of biomolecular condensates in establishing microenvironments characteristic of HCC biology. It is well established that P62 can protect ROS-rich HCC-initiating cells from oxidative stress-induced cell death by activating the Nuclear Factor Erythroid 2-Related Factor 2 (Nrf2) and the mechanistic Target of Rapamycin Complex 1 (mTORC1) pathways thereby contributing to its persistence-elevated levels of p62 have been observed in HCC progenitor cells and are commonly found in most chronic liver diseases that progress to HCC. Now it is increasingly realized that biological condensates formed by P62 also contribute to this process. P62 are critical components of Mallory-Denk bodies and intracellular hyaline bodies, which are characteristic of chronic liver diseases and significantly increase the risk of HCC (Büscher et al., 2022; Kurusu et al., 2023). Vault RNA (vtRNA), a component of the vault complex has been found to negatively regulate p62-body formation by binding p62 via its Phox1 and Bem1p (PB1) domain or adjacent region to inhibit its polymerization (Tanaka et al., 2009). Both Major Vault Protein (MVP) and Neighbour of BRCA1 gene 1 (NBR1) are able to colocalize with p62-positive Mallory-Denk bodies. These structures are prominent in steatohepatitis and HCC, and their formation depends on the phase separation of p62. Moreover, Modulator of Apoptosis 1 (MOAP-1) plays a vital role in downregulating p62 bodies and Nrf2 activity in livers exposed to the carcinogen Diethylnitrosamine (DEN). This regulation significantly reduces the susceptibility to liver tumourigenesis induced by DEN exposure. It is through the regulation of autophagy and ferroptosis that LLPS is involved in cellular homeostasis and quality control, and the abnormal mechanism of LLPS leads to the occurrence of HCC (Kurusu et al., 2023).

### Condensate mediated dysregulation of epigenetics

Recent advancements in understanding protein synthesis have shed light on the involvement and regulatory role of LLPS in this crucial biological process. Abnormalities in this mechanism, such as the mislocalisation of FUS and dysfunctional phase separation within the RNA Binding Protein (RBP) network, have been found to be associated with LLPS and have been implicated in disrupting neuronal function. These disruptions, caused by a misregulation of precise translational requirements, may contribute to the development of Amyotrophic Lateral Sclerosis (ALS) and impact the survival of motor neurons. Moreover, specific mutations in an RBP can induce the formation of condensates that sequester other RBPs, thereby interfering with essential biological functions in protein translation regulation (Birsa et al., 2021).

Biomolecular condensates have also been identified as a major regulator in the progression and metastasis of HCC by influencing mRNA degradation. One key player in this process is YTHDF2, a reader protein that recognizes N6-methyladenosine (m6A) modification on mRNA (Wang et al., 2014; Shi et al., 2019). YTHDF2 controls mRNA decay by guiding it to membrane-less cytoplasmic P granules for its destruction (Wang et al., 2014). The deficiency of YTHDF2 promotes HCC growth and serves as a prognostic marker for HCC patients. In cell models, manipulating the levels of YTHDF2 in liver cancer cells, either through knockdown or overexpression, leads to changes in OCT4 protein expression. This occurs because the 5′-untranslated region (UTR) of OCT4 mRNA can be modified with m6A marks. These alterations contribute to the generation of cancer stem cells and the transformation of cancer cells, influencing the HCC phenotype (Zhang C. et al., 2020). Recent studies have now demonstrated that YTHDF2 undergoes phase separation, a process that is promoted by m6A-modified RNA (Wang et al., 2020). The ability for YTHDF2 to form condensate may provide an additional explanation for the control of m6A methylations of OCT4 mRNA by YTHDF2, in turns its effect on promoting the cancer stem cell (CSC) liver phenotype and facilitating cancer metastasis (Zhang C. et al., 2020; Wang and Lu, 2021).

Stress granules (SGs) are specialized membrane-less ribonucleoprotein (RNP) condensates that form in the cytoplasm in response to environmental stress (Namkoong et al., 2018). Under stress conditions, these granules are promoted by the circular RNA circVAMP3, which plays a crucial role in suppressing tumour cell proliferation and metastasis. In the context of HCC, circVAMP3 co-localizes with CAPRIN1, and c-Myc within SGs, this not only promotes further CAPRIN1 and stress granule phase separation, but also inhibits c-Myc translation (Chen et al., 2022). The reduction in the levels of Myc protein thus leads to the suppression of proliferation and metastasis in HCC. This mechanism emphasizes the involvement of LLPS in the post-transcriptional regulation of RNA molecules in HCC (Liu B. et al., 2023).

RNAs can drive LLPS via electrostatic interactions (Tsoi et al., 2021; Tong et al., 2022). Additionally, through repeated intermolecular base pairing, they can achieve multivalency, and lead to cluster formation, thus they modulate phase behaviour (Tong et al., 2022). Long non-coding RNAs (lncRNAs), which are non-protein-coding RNA molecules over 200 nucleotides long, constitute a major part of human genome transcripts (Wei et al., 2023). lncRNAs exert their influence through interactions with DNA, proteins, and other RNAs. Furthermore, the LLPS-related lncRNAs signature may help assess prognosis and predict treatment efficacy in clinical settings (Hashemi et al., 2022). Specifically, the lncRNA ZNF32-AS2 is involved in regulating liver cancer cell proliferation, mobility, sorafenib resistance, and tumour growth, making it a potential biomarker for HCC. Therefore, emphasis should be laid on prognostic importance of LLPS-related lncRNAs in hepatocellular HCC (Huo et al., 2017; Peng et al., 2024).

## Overview of LLPS in HCC cell nucleus

In this comprehensive exploration, we delve into the complex dynamics of intracellular signalling and its implications in HCC cell nucleus. Specifically, we focus on the role of LLPS in the deregulation of transcription within the nucleus. Then, we investigate the important roles of super-enhancers and the phase separation ability of YAP in gene transcription. Additionally, we discuss the impact of proteins like BRD4 and Pinin on nuclear localisation and transcriptional regulation, which are intricately linked to oncogenesis. Moreover, we delve into the complexities of epigenetic dysregulation mediated by LLPS.

### LLPS and dysregulation of transcription

**FIGURE 2:**
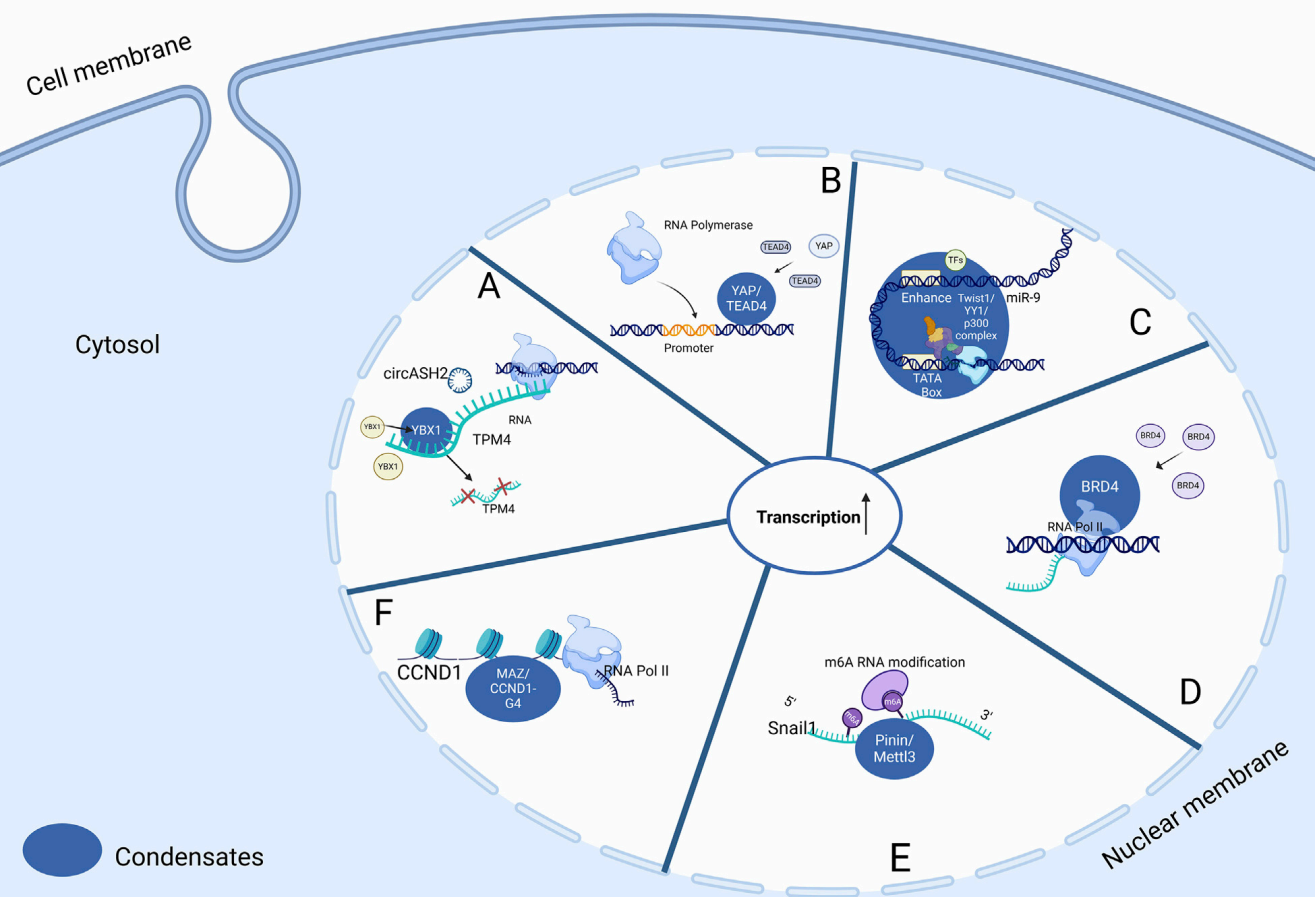
LLPS and transcriptional regulation in the nucleus of HCC cells. **(A)** LLPS of nuclear YBX1 enhanced by circASH2 augments TPM4 transcripts decay. **(B)** Initiation of transcriptional start sites by YAP and TEAD4 is facilitated by LLPS, which promotes HCC cell proliferation. **(C)** Formation of condensate between SE elements at the miR-9 super-enhancer site promotes its expression. **(D)** BRD4 can form discrete condensates in chromatin to facilitate active gene transcription. **(E)** Pinin augments Mettl3 localisation within nuclear speckles (a form of condensates), to promote m6A modification, leading to increased snail1 expression. **(F)** G4s recruit MAZ to the CCND1 promoter and facilitate the motility in MAZ condensates that compartmentalize coactivators to activate CCND1 expression. Abbreviations: TEAD1: TEA Domain transcription factor 1, YAP/TEAD4: Yes-Associated Protein/TEA Domain transcription factor 4, TFAM: Transcription Factor A, Mitochondrial, circASH2L: circular RNA ASH2L, YBX1: Y-box Binding protein 1, TPM4: Tropomyosin 4, BRD4: Bromodomain containing 4, CCND1: Cyclin D1, CCND1-G4: Cyclin D1 G-quadruplex, MAZ: MYC Associated Zinc finger protein, m6A: N6-methyladenosine, Mettl3: Methyltransferase 3.

Transcription factors (TFs) may exhibit a predisposition to undergo LLPS due to the presence of intrinsically disordered regions (IDRs). Disease-associated repeat expansions in various TFs were found to modify their phase separation behaviours in a similar way. The transcription factors HOXA13, HOXD13, RUNX2, and TBP possess these regions, and it is within the LLPS bodies they form that these transcriptional factors interact with the transcriptional machinery (Boija et al., 2018; Sabari et al., 2018). Regulation of biological condensates within the nucleus involves interactions with nuclear circular RNA. Condensates formed by Y-box binding protein 1 (YBX1) and heterogeneous nuclear ribonucleoproteins (hnRNPs), involved in pre-mRNA splicing and mRNA decay regulation, is subject to modulation by the presence of circASH2, a nuclear circular RNA. (Figure 2A). This interaction enhances condensate formation and leads to an accelerated decay of TPM4 transcripts (Liu B. et al., 2023).

Proteins containing a coiled-coil domain are also prone to phase separation. One example is YAP. It shows two regions predicted to be IDR, including the TEA Domain Transcription Factor 1 (TEAD) domain, the CC domain, and the TAD domain. Nuclear-active YAP forms transcription sites via LLPS, regulating target gene transcription and promoting hepatoblastoma (HB) cell proliferation (Chen et al., 2019; Mao et al., 2023) (Figure 2B).

Super-enhancers (SEs), the dense clusters of enhancer elements, have important functions in regulating tissue-specific gene transcription through its ability to recruit specific TFs (Mansour et al., 2014; Boija et al., 2018). LLPS can indirectly regulate SE elements: Twist1, a transcription factor which significantly contributes to tumour metastasis, can complex with YY1 TF and p300 cofactor to form condensates at the miR-9 super-enhancer site to promote miR-9 expression. In HCC, elevated miR-9 expression is associated with epithelial-mesenchymal transition (EMT), leading to a more invasive phenotype (Meng et al., 2023) (Figure 2C).

Additionally, the short isoform of BRD4S forms discrete condensates in chromatin, facilitating active gene transcription. Studies reveal a robust correlation between endogenous BRD4 puncta in the nuclei of liver cancer cell and BRD4S and total BRD4 levels, but not with the long isoform of BRD4L (Han et al., 2020; Shi et al., 2023) (Figure 2D).

Pinin, characterized as a multifunctional protein, exhibits dual localisation within the cellular architecture, being found both at the desmosome and in the nucleus (Ouyang, 1999). Recent studies have elucidated Pinin’s nuclear localisation, particularly in nuclear speckles, and its significant role in transcriptional regulatory processes (Brandner et al., 1998). These processes encompass alternative splicing and transcriptional modulation, with a notable association with oncogenesis (Costa et al., 2006). Pinin is hypothesized to act as a scaffold protein in the interaction between Pinin and Mettl3, potentially influencing m6A RNA modification levels (Figure 2E). This scaffolding role enhances the localisation of Mettl3 within nuclear speckles, thereby promoting m6A modification levels and the upregulation of snail1, a crucial transcription factor implicated in EMT (Wang et al., 2014; Qiao et al., 2021).

Four-stranded G-quadruplex (G4) structures form through self-recognition of guanines into stacked tetrads. The initial detection of four-stranded G-quadruplex (G4) structures emerging from natural sequences originated from the observation of higher-order secondary structures of nucleic acids *in vitro*. These structures arose from oligonucleotides bearing resemblance to G-rich sequences found in the immunoglobulin switch region (Varshney et al., 2020). G4 can promote molecular motility in MAZ-containing condensates. Recent research revealed that G4s recruit MAZ to the CCND1 promoter and facilitate the molecular motility in MAZ condensates that compartmentalize coactivators to activate CCND1 expression and subsequently exacerbate hepatocellular carcinogenesis (Wang et al., 2024) (Figure 2F).

### LLPS and cellular quality control

SURF6, an intrinsically disordered protein, plays a crucial role in ribosome biogenesis and cellular proliferation, thereby regulating the process of ribosome biogenesis. It co-localizes with Nucleophosmin (NPM1), a protein that confers liquid-like characteristics to the granular component of the nucleolus through phase separation. SURF6 is implicated in regulating the cell cycle and is observed to be elevated in liver cancers, aligning with the overexpression of nucleolar constitutive proteins in these malignancies (Ferrolino et al., 2018).

### LLPS and abnormal activation of oncogenes and tumour suppressor genes

Oncogene-Related Condensates: The dysregulation and heightened expression of MYC transcription factors, including MYC itself, are implicated in the majority of human cancers (Yang et al., 2022). In the context of HCC, the circular RNA circVAMP3 has been identified as playing a role in the abnormal activation of MYC through the formation of biomolecular condensates. CircVAMP3 promotes the assembly of condensates containing MYC and its cofactors, leading to enhanced transcriptional activity and HCC progression. Also, LLPS of RIα, a PKA regulatory subunit, controls cAMP compartmentalisation as well as oncogenic signalling. The loss of RIα LLPS in cells promotes cell proliferation and induces cell transformation in liver cancer (Chen et al., 2022).

Tumour Suppressor-Related Condensates: The tumour suppressor protein p53, known for its role in maintaining genomic stability and preventing tumour formation, also undergoes LLPS in the nucleus. Phase separation of p53 facilitates its interactions with various cofactors and target genes, enabling its tumour suppressive functions. Disruption of p53 phase separation impairs its transcriptional activity and contributes to the development of HCC (Kamagata et al., 2020; Xiao et al., 2023).

### LLPS and epigenetic dysregulation

Nuclear bodies, such as nucleoli and promyelocytic leukaemia (PML) bodies are membraneless organelles formed through LLPS. These structures play crucial a role in various nuclear processes, including transcriptional regulation, RNA processing, and DNA repair. Disruption of phase separation leads to nuclear body dissolution, giving rise to genomic instability and contribute to HCC development (Sawyer et al., 2019).

The MLL1/KMT2A protein, a histone methyltransferase implicated in gene activation, undergoes LLPS in the nucleus. MLL1/KMT2A concentrates into liquid-like droplets, facilitating its interactions with chromatin and promoting gene expression. Dysregulation of phase separation can disrupt its normal function and contribute to epigenetic dysregulation in HCC (Sha et al., 2023).

In summary, the dysregulation of transcription through LLPS within the nucleus plays a significant role in HCC development. The phase separation of transcription factors, super-enhancers, and proteins like YAP, BRD4, Pinin, and p53 contribute to altered gene expression patterns and cellular phenotypes in HCC. Additionally, cellular quality control mechanisms, abnormal activation of oncogenes and tumour suppressor genes, and epigenetic dysregulation mediated by LLPS further contribute to HCC progression. Understanding the intricate dynamics of LLPS in the nucleus provides valuable insights into the molecular mechanisms underlying HCC and may pave the way for the development of novel therapeutic strategies.

## LLPS in both the nucleus and cytoplasm of HCC cells

We discuss the tumourigenesis of LLPS in hepatoma cells, focusing on both the nucleus and the cytoplasm. Specifically, we explore the formation of biomolecular condensates involving STAT3 in response to IL-6 activation. These condensates have been observed in both the nucleus and the cytoplasm of hepatoma cells. In hepatocytes with wild-type p53, exposure to IL-6 leads to a reduction in STAT3 immunostaining in the nucleus, despite the presence of intact STAT3 protein as confirmed by Western blotting (Rayanade et al., 1997). This phenomenon, referred to as “STAT masking,” is now understood as a cytoplasmic phase transition of STAT3 into biomolecular condensate structures. These structures are not detectable by anti-STAT3 antibodies (Sehgal, 2019). Importantly, this process is transient and can be inhibited by proteasomal inhibitors like lactacystin and MG-132 (Sehgal, 2019; Sehgal, 2022).

## Potential strategies to target HCC by disrupting pathogenic LLPS

**FIGURE 3:**
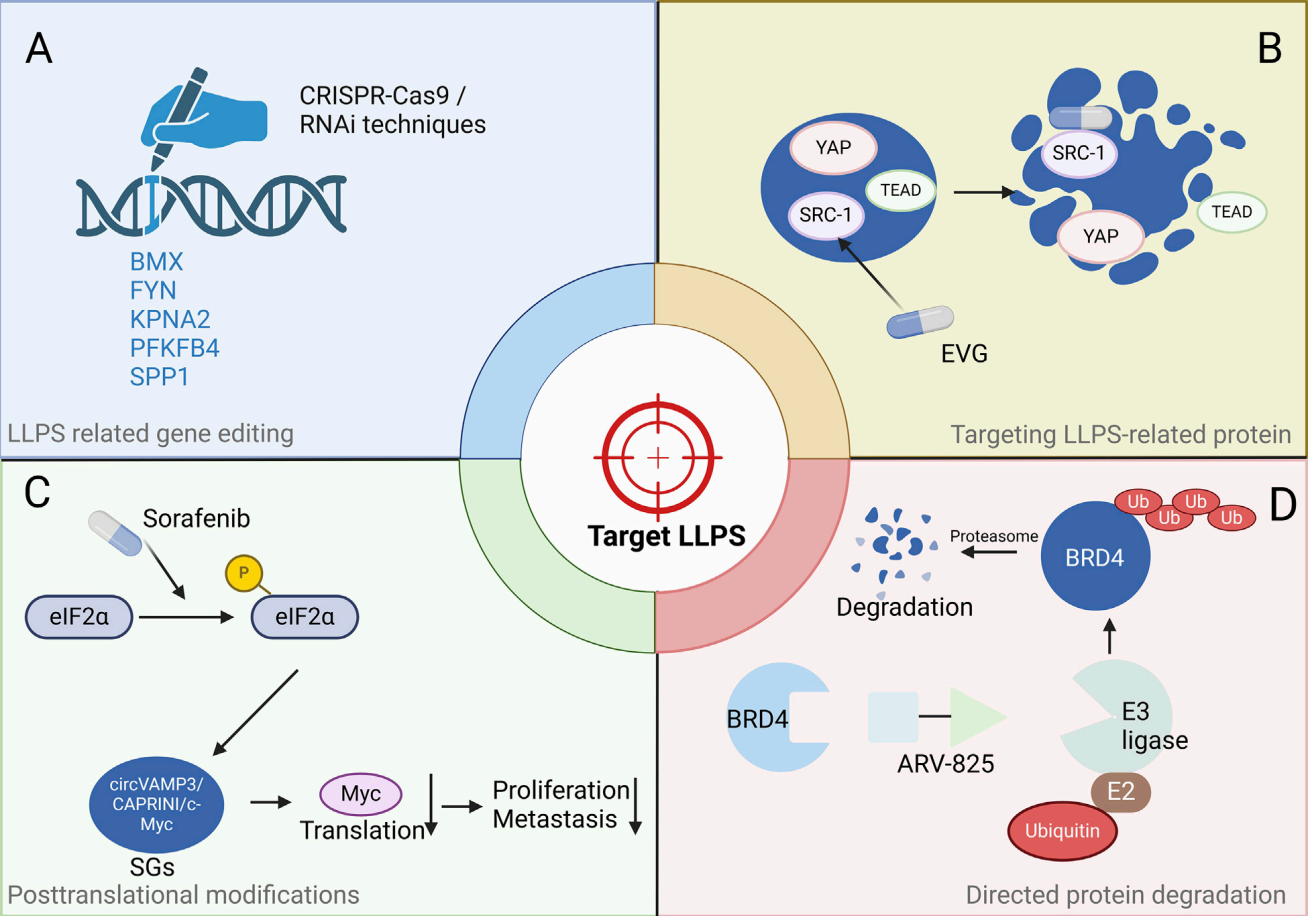
Potential strategies to target HCC by disrupting LLPS. **(A)** Targeting genetic phase separation at the genetic level, e.g., using CRISPR-Cas9 gene editing or RNA interference techniques. **(B)** Inhibitors targeting LLPS-related proteins include various approaches such as small molecules, peptides, and aptamers, e.g., Elvitegravir directly bind to SRC1 and effectively inhibit YAP oncogene transcription by disrupting condensates. **(C)** The drug that modifies a protein post-translationally, e.g., Sorafenib induces SG formation, leading to a reduction in the levels of Myc protein. **(D)** Directed protein degradation by PROTAC, e.g., ARV-825 recruits BRD4 to the E3 ubiquitin ligase cereblon, resulting in the downregulation of MYC expression. Abbreviations: eIF2α: eukaryotic Initiation Factor 2α, circVAMP3: circular Vesicle Associated Membrane Protein 3, SGs: Stress granules, YAP: Yes-Associated Protein, TEAD: TEA Domain transcription factor, SRC-1: Steroid Receptor Coactivator-1, EVG: Elvitegravir, BRD4: Bromodomain containing 4, E3 ligase: E3 ubiquitin ligase, E2: E2 ubiquitin-conjugating enzyme, Ub: Ubiquitin. Note: Figures were created using BioRender.

It is increasingly recognized that targeting LLPS holds promise as an effective strategy for therapeutic intervention in cancer. Several small molecules have been identified that impact LLPS and related processes. The study identifies gene modules related to LLPS that correlate with the tumour grade of HCC. The Liquid-Liquid Phase Separation Risk Index (LLPSRI) emerges as a promising prognostic marker for HCC, capable of distinguishing patients with low or high risk of adverse survival outcomes (Fang et al., 2021). Through rigorous analysis, 43 LLPS-related genes were found to significantly impact the overall survival (OS) of HCC patients. Among these, five genes—BMX, FYN, KPNA2, PFKFB4, and SPP1—were utilized to create a prognostic risk score signature, highlighting their potential as therapeutic targets for HCC treatment (Fang et al., 2021; Lai et al., 2023). We propose that targeting genetic phase separation using CRISPR-Cas9 gene editing or RNA interference (RNAi) techniques will be a clinically effective attempt and a novel therapeutic strategy in HCC (Figure 3A).

The tumour suppressor protein p53 can form aggregates, with disease-relevant mutants accelerating this process to form aggregates, oligomers, and even fibrils. These structures can impair the transcriptional function of p53. The YAP oncogene acts upstream to inhibit apoptosis and promote cell growth, often through negative interaction with p53 tumour suppressor (Vousden and Prives, 2009; Kamagata et al., 2020). The highly disordered steroid receptor coactivator 1 (SRC1) colocalizes with YAP-transcriptional enhancer factor domain family member TEAD in phase-separated transcriptional condensates. The anti-HIV drug Elvitegravir (EVG) directly binds to SRC1 and effectively inhibits YAP oncogene transcription by disrupting SRC1 LLPS in SRC1/YAP/TEAD condensates (Zhu et al., 2021; Liao et al., 2022) (Figure 3B). Thus, EVG presents itself as a promising treatment option for HCC, featuring a pathway akin to that of YAP.

SGs are involved in pathogenesis of cancers, just as mentioned. CircVAMP3 co-localizes with CAPRIN1, and c-Myc within SGs in HCC. Additionally, small molecules can affect SG dynamics, including assembly, disassembly, maintenance and clearance (Chen et al., 2022). Thus, targeting SGs is a potential therapeutic strategy. The major SG formation-regulating signalling pathways include the eIF4F and eIF2α pathways, and the mammalian target of rapamycin (mTOR) is also involved in signalling. The Raf1/Mek/Erk kinase inhibitor sorafenib is approved for advanced HCC, and it’s noted that the formation of SGs in sorafenib-treated HCC cells correlates with inhibition of translation initiation (Tong et al., 2022). Sorafenib induces SG formation in various cancer cells via PERK-mediated eIF2α phosphorylation (Figure 3C). This highlights the potential of directly targeting LLPS as a new avenue for cancer therapy.

A promising strategy to target condensates in HCC involves the use of cell-penetrating peptides, which can selectively disrupt phase-separated condensates via IDR and DNA-binding domains (DBD). The designed peptide DP6 binds to TP53, inhibiting its DNA binding and suppressing its tumour suppressor activity (Gabizon et al., 2012). Another peptide, ReACp53, selectively targets mutant p53 to rescues p53 transcription of target genes and restores apoptosis, but has no effect on wild type p53, offering a precise treatment in cancer (Soragni et al., 2016). In addition, Kang et al. designed the 2142-R8 blocking peptide, which disrupts the KAT8-IRF1 condensate, inhibits PD-L1 expression and enhances antitumor immunity both *in vitro* and *in vivo* (Wu et al., 2023). Therefore, based on the mechanisms related LLPS involved in HCC progression outlined in this study (Table 1), specific blocking peptides can be designed to target and disrupt these condensates.

Recent studies have highlighted that aptamers and homologous double-stranded DNA can organize TP53 into amyloid-like structures. Binding to a consensus DNA sequence stabilizes the TP53 core domain (p53C) and prevents misfolding, an effect tied to DNA-binding affinity (Ishimaru et al., 2009). Aptamers are nucleic acids with high specificity and affinity for single protein targets, uniquely capable of binding with varying affinities to different conformations of the same protein (Macedo and Cordeiro, 2017). Similarly, they can be applied as potential tools to target phase-separated proteins, offering a novel approach to modulate LLPS mechanisms in HCC and serve as a promising therapeutic strategy. Thus, targeting LLPS in HCC can involve a variety of approaches, including the use of small molecules, cell-penetrating peptides, aptamers, and other inhibitors to disrupt condensates in HCC.

Disrupting condensate formation through the manipulation of IDRs or physicochemical properties represents a promising method. Emerging evidence suggests that small molecules have the ability to interact with IDRs of transcription factors—such as TAF2, MYC, c-FOS, p53, and EWS—to inhibit malignant cell transformation (Ren et al., 2022). Research found that ABCF1 interacts with closed circular DNA (cccDNA) to form phase-separated condensates via the poly-glutamine (PolyQ) of N-terminal IDR. ABCF1 acts as an antiviral restriction factor of HBV cccDNA by phase-separation-driven innate immune signalling and transcription inhibition (Ren et al., 2023). We can delay and even prevent the progression of HCC by interfering with IDR to prevent the replication of HBV.

Recently, protein degradation mediated by proteolysis targeting chimeras (PROTACs) represents itself as the latest progress in the treatment of prostate cancer (Stone, 2023). PROTACs are heterobifunctional compounds composed of two ligands linked together, enabling the simultaneous recruitment of a protein of interest (POI) and an E3-ubiquitin ligase. This dual action induces ubiquitination and subsequent proteolysis of the POI. PROTACs leverage the cell’s inherent protein degradation machinery to selectively remove specific target proteins from cells (Li et al., 2022). Lu et al. designed a heterobifunctional PROTAC, ARV-825, which can lead to efficient and prolonged degradation of BRD4 in BL cell lines by recruiting BRD4 to the E3 ubiquitin ligase Cereblon and thus downregulating the expression of MYC (Lu et al., 2015). Applying the same principle, targeting the phase separation of BRD4 with the BRD4 degrader ARV-825 (Figure 3D), particularly focusing on its short isoform (BRD4S), known for forming distinct condensates in chromatin within liver cancer cells, has proven to be an efficacious approach for targeting HCC (Shi et al., 2023).

## Discussion

In summary, we have discussed the intricate interplay between LLPS and HCC, with a specific focus on its ramifications within the cell, encompassing both cytoplasmic and nuclear domains. This includes how LLPS disrupts certain cell signalling pathways like MAPK, cAMP-dependent, and Hippo, which contributes to HCC development. Additionally, our examination has scrutinized the role of LLPS in regulating cell viability via mechanisms such as Ferroptosis and autophagy. We have explored its impact on gene expression and RNA dynamics, as well as its influence on critical cellular processes including transcription and nuclear DNA organization. These insights could help understand HCC better and find new treatment. Furthermore, we have discussed how a specific long non-coding RNA (FASA) interacts with PRDX1, affecting its function and altering the balance of ROS through LLPS. This process holds significant importance in elucidating cellular iron homeostasis, particularly in how LLPS governs the degradation of Ferritin, thereby influencing ferroptosis—a type of cell death pivotal in cancer therapy. In particular, the lncRNA URB1-AS1’s modulation of Ferritin via LLPS can lead to reduced cellular iron levels, thereby impacting ferroptosis and, consequently, influencing the outcomes of cancer treatment. Finally, while most LLPS research in HCC focuses on the nucleus and cytoplasm, studying how it affects cell membrane receptors could offer valuable insights into the underlying mechanisms of HCC.

Then, we focused on potential novel treatment strategies for HCC targeting biomolecular condensates and disrupting LLPS. We highlight the identification of LLPS-related gene modules linked to HCC tumour grades and introduce the LLPSRI as a promising prognostic marker. The study identifies 43 LLPS-related genes significantly affecting patient survival, with five genes forming a prognostic risk score signature. It suggests CRISPR-Cas9 and RNAi as potential methods for disrupting genetic phase separation in HCC treatment. Additionally, the article discusses the role of SGs and IDRs in cancer pathogenesis, pointing out specific small molecules and drugs like Sorafenib and Elvitegravir that impact LLPS dynamics and offer therapeutic benefits. It also mentions the use of PROTACs for protein degradation, providing a comprehensive overview of emerging therapeutic strategies targeting LLPS in HCC.

Existing studies have demonstrated the impact of LLPS on various signalling pathways, including MAPK pathways, the cAMP-dependent pathway, and the Hippo signalling pathway. By specifically disrupting phase-separated condensates, we can modulate these pathways. For instance, disrupting EML4-ALK in the MAPK pathway and RIα in the cAMP-dependent pathway can be effective. Additionally, targeting the Laforin-Mst1/2 complex to inhibit the Hippo signalling pathway may impede liver tumour initiation. Although technologies like PROTAC are addressing the challenge of targeting, the precise targeting and degradation of phase-separated condensates remain to be unsolved hurdles. Furthermore, based on the fact that p62 is a critical component of Mallory-Denk bodies and intracellular hyaline bodies in autophagy as mentioned above, it is worth considering that modulating the phase separation involving p62 could have a significant impact on HCC treatment.

Research and development efforts are currently underway for drugs leveraging phase separation technology. To date, five companies globally are utilizing this technology for novel drug development, with a notable example being Dewpoint Therapeutics. Dewpoint Therapeutics aims to address related diseases by screening small molecule compounds that regulate cell phase separation and restore the liquid morphology of membrane-free organelles. Our review provides sufficient evidence for the involvement of phase separation in HCC, and we hope that more clinical studies will focus on targeted investigations and drug trials in HCC, such as BRD4, which forms distinct condensates on chromatin, facilitating active gene transcription in liver cancer cell nuclei. By combining the dynamics of protein BRD4 condensates with PROTAC technology, we anticipate promising results from future experimental studies.

The DNA damage response is crucial for maintaining genomic stability, and recent research shows that the repair centre is characterized as a condensate formed by LLPS of key DSB repair factors. DNA damage repair mechanisms associated with LLPS are crucial, yet there is a paucity of cancer-related phase separation experiments in HCC currently. Further research in this direction holds significant promise for enhancing our understanding of HCC mechanisms. The advancement of targeted phase separation studies is in its nascent phase, and effectively applying phase separation targeting technology to specific targets poses a significant challenge. Nevertheless, ongoing endeavours are directed towards drug development based on phase separation, particularly for nervous system diseases and cancer. With publication of this review, we anticipate further exploration of phase separation drugs and clinical studies related to HCC, which will pave the way for a new era in liver cancer treatment. Further research is needed to elucidate the precise mechanisms underlying phase separation in HCC and its functional implications in cellular processes and disease.
